# Partial Discharge Detection from Large Motor Stator Slots Using EFPI Sensors

**DOI:** 10.3390/s25020357

**Published:** 2025-01-09

**Authors:** Jinlong Wang, Weizhong Sun, Jun Zhou, Lei Wang, Lianfei Chen, Pengcheng Chen, Qichao Chen, Weichao Zhang

**Affiliations:** 1 Yunnan Electric Test & Research Institute Group Co., Ltd., Kunming 650214, China; wangjinlong@dlsyyjy.yn.csg.cn (J.W.); sunweizhong@dlsyyjy.yn.csg.cn (W.S.); zhoujun@dlsyyjy.yn.csg.cn (J.Z.); wanglei@dlsyyjy.yn.csg.cn (L.W.); chenlianfei@dlsyyjy.yn.csg.cn (L.C.); chenpengcheng@dlsyyjy.yn.csg.cn (P.C.); 2 School of Electrical and Electronic Engineering, Harbin University of Science and Technology, Harbin 150080, China

**Keywords:** EFPI sensor, fluid–structure interaction, partial discharge ultrasonic signals, acoustic field distribution, PD source localization

## Abstract

This study addresses the challenges of electromagnetic interference and unstable signal transmission encountered by traditional sensors in detecting partial discharge (PD) within stator slots of large motors. A novel Extrinsic Fabry–Perot Interferometer (EFPI) sensor with a vibration-coupling air gap was designed to enhance the narrowband resonant detection sensitivity for PD ultrasonic signals by optimizing the diaphragm structure and coupling interface. The sensor features a quartz diaphragm with a thickness of 20 μM, an effective constrained radius of 0.9 mm, a vibration-coupling air gap depth of 100 μM, and a first-order natural resonant frequency of 66 kHz. Simulation and experimental analyses revealed the distribution characteristics and propagation paths of ultrasonic signals within stator slots. The results demonstrate that the EFPI sensor effectively detects PD ultrasonic signals at its resonant frequency of 66 kHz with a localization error of less than 5 mm, meeting engineering requirements. This study provides theoretical and practical insights into the efficient detection and precise localization of insulation faults in large motor stators.

## 1. Introduction

As the rated capacity and voltage levels of generators continue to increase, the demands for insulation reliability in large generators have become increasingly stringent. Large motors are extensively utilized in various critical sectors such as power plants, petrochemicals, mining, and manufacturing, and operational stability is vital for both productivity and daily life [1]. Any failure resulting in downtime can have significant repercussions. However, the degradation and damage to the motor stator insulation system can severely disrupt normal operation, potentially leading to unexpected equipment failures and outages. During operation, the stator windings of the generator are subjected to long-term electromechanical, thermal, and mechanical stresses. With the electric field concentrating at the ends, the ends of generator windings represent a weak point in insulation that is more susceptible to partial discharge (PD). Partial discharge is one of the primary indicators of insulation deterioration [2]. PD refers to localized electrical discharges that occur within or on the surface of electrical insulation materials. These discharges typically arise in areas with insulation defects, air gaps, or regions of intense electric field. Although the energy involved in the partial discharge phenomena is relatively small, its cumulative effects over time can gradually degrade insulation materials, ultimately leading to insulation breakdown and equipment failure [3]. Therefore, timely detection and monitoring of partial discharge are crucial for ensuring the safe operation of large electrical machines.

Currently, partial discharge (PD) detection methods can be broadly categorized into electrical and non-electrical techniques. Among the electrical methods, the pulse current method and radio frequency (RF) signal detection method are widely used for diagnosing electrical equipment such as motors [4,5], transformers [6,7], and gas-insulated switchgear (GIS) [8]. The pulse current method suffers from poor on-site applicability due to the large size of detection instruments, particularly for routine inspection tasks in industrial applications. In contrast, the radio frequency (RF) signal detection method has garnered more attention and research because of its smaller instrument size, simplicity of operation, and clear advantages in usability. This is especially true in recent years, as the installed capacity of substations, power plants, and other power sectors has increased, along with the number of associated power facilities. Consequently, the surge in inspection workloads has driven researchers to devote significant effort to the study and development of RF sensors, particularly in enhancing the detection sensitivity of these sensors. For example, the authors of [9] studied the deployment position of sensing units and proposed a passive wireless RF sensor embedded in the observation window of GIS. By employing a multi-resonance approach, the detection sensitivity was significantly enhanced. The authors of [10] designed a mechanical device equipped with RF sensors, which, in combination with a controllable mechanical vehicle, could synchronize with motors of varying horizontal diameters, thereby improving the reliability and detection sensitivity of on-site PD detection systems. The authors of [11] focused on the RF sensor itself, developing an electrically small hybrid helical antenna. The incorporation of a ring resonator enhanced the low-frequency gain, resulting in improved average received power and power spectral density. The authors of [12], building on the detection of PD using flexible composite helical antennas, applied a residual shrinkage network to improve the accuracy of PD pattern recognition. Validation results showed that even in high-noise environments, the recognition accuracy exceeded 90%. Nevertheless, as the sensitivity of sensors continues to improve, the RF signal detection method faces increasingly pronounced challenges in complex electromagnetic environments, particularly in the operational settings of large generators. Additionally, RF signal detection struggles with the critical issue of pinpointing the exact location of PD occurrences.

Non-electrical detection methods primarily include optical signal detection and ultrasonic signal detection. Optical signal detection is mainly used for monitoring power equipment located in open areas, such as transformer bushings, dry-type reactors, and overhead lines [13,14,15]. However, it faces challenges in detecting partial discharge (PD) within the stator insulation of motors. In contrast, ultrasonic signal detection has distinct advantages, including strong immunity to electromagnetic interference, a detection frequency band far from the low-frequency vibration signals of power equipment, and high precision in PD location determination [16,17]. Nevertheless, the application of ultrasonic PD detection technology for motor stator PD detection is rarely reported in existing research. This is primarily because traditional piezoelectric ceramic (PZT) acoustic emission sensors exhibit low detection sensitivity, require active on-site detection, and rely on electrical signal transmission, making them susceptible to electromagnetic interference from the operational environment of motor equipment. With the rapid development of advanced optical fiber sensing technology, optical fiber acoustic emission sensors for PD detection have demonstrated significantly superior sensitivity compared to PZT sensors in detecting ultrasonic signals from PD in various power equipment. Additionally, optical fiber sensors enable passive on-site detection and inherently resist electromagnetic interference, which has drawn substantial attention and research interest toward optical fiber acoustic emission sensors for PD detection [18,19,20].

Among various optical fiber ultrasonic sensors for partial discharge (PD) detection, the Extrinsic Fabry–Perot Interferometer (EFPI) sensor has garnered significant attention due to its high detection sensitivity, simple demodulation system, and compact structure. In the early 21st century, Anbo Wang and colleagues utilized quartz as an optoacoustic transduction element to design and fabricate a quartz diaphragm with a thickness of 125 μM and a diameter of 2.5 mm. Using this diaphragm, they developed an EFPI sensor with a cavity length of 15.6 μM, achieving a sensitivity of 3.5 nm/kPa and a resolution of 10 Pa [21]. Similarly, Wang Xiaodong et al. employed Micro-Electro-Mechanical System (MEMS) technology to design and fabricate a silicon diaphragm with a thickness of 25 μM and a side length of 2 mm as an optoacoustic transduction element. The EFPI sensor had a cavity length of 90 μM, and through multi-cycle demodulation, each interference fringe corresponded to a sound pressure of 552 Pa, with a minimum detectable sound pressure of 2.8 Pa [22]. In 2018, Wang Peng and colleagues applied an array of four EFPI sensors to detect PD ultrasonic signals in transformer oil. They employed the Two-Sided Correlation Transformation (TCT) algorithm to correct positional errors of the sensor array elements, enhancing measurement accuracy [23]. In 2020, Li Haoyong utilized MEMS technology to fabricate a silicon diaphragm EFPI sensor with a cross-support beam structure. The diaphragm thickness was 5 μM, and at the resonant frequency, the detection sensitivity was −10 dB re. 1 V/Pa [24]. Subsequently, using a Fresnel Zone Phase Correcting Plate (FZP) structure, the researcher optimized the sensor’s detection sensitivity, improving it from −19.8 dB re. 1 V/Pa to −12.4 dB re. 1 V/Pa at the resonant frequency [25]. Chaofei Gao et al. conducted extensive studies using EFPI sensors to detect PD ultrasonic signals in power equipment such as transformers. Their results demonstrated that EFPI sensors enable precise PD localization and pattern recognition [26]. Weichao Zhang and colleagues optimized the detection sensitivity of EFPI sensors by analyzing the spectral characteristics of PD ultrasonic signals and conducted in-depth studies on the comprehensive detection properties of EFPI-based single-arm acoustic emission sensors [27,28]. These advancements have significantly accelerated research on EFPI sensors for detecting PD ultrasonic signals in power equipment, laying a solid foundation for their practical engineering applications. However, despite these achievements, the research predominantly focuses on PD ultrasonic signal detection in fluid insulation media, where sound signals are coupled to the EFPI sensor through the fluid medium. There is a notable lack of research on coupling EFPI sensors for PD ultrasonic signal detection on solid media surfaces, which significantly restricts their application scenarios in engineering practices. Moreover, studies on the distribution and propagation characteristics of PD ultrasonic signals in large motor stators are scarce. Given the complex structure of large motor stators and the diversity of propagation interfaces, challenges remain in selecting detection points for PD ultrasonic signals and determining the installation and coupling methods for sensors. Additionally, a critical technological gap exists in enabling the EFPI sensor’s diaphragm, as an acoustic-sensitive element, to detect PD ultrasonic signals at gas-solid composite interfaces inside large motor stators.

This paper presents a method for detecting partial discharge ultrasound in generator stator bars using EFPI sensors. It elucidates the principles and characteristics of EFPI partial discharge detection. Based on simulation and experimental analysis, the acoustic coupling characteristics of the gas-solid composite interface sensor are examined. A structure for the EFPI sensor suitable for detecting partial discharge in large motor stator bars is designed. Furthermore, the study investigates the distribution and propagation characteristics of partial discharge ultrasound signals in large motor stators, alongside research on the detection and localization of partial discharges in stator slots. This work provides both theoretical and practical foundations for ultrasound detection of partial discharge in generator stator windings.

## 2. Principle of EFPI Sensor Detection and Analysis of Acoustic Coupling Characteristics

### 2.1. Principle of Sensor Detection

**Figure 1:**
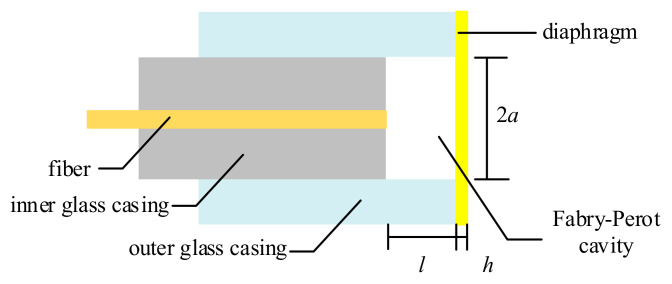
Schematic diagram of EFPI sensor structure.

The EFPI sensor detects partial discharge ultrasonic signals by using its front diaphragm as an acousto-optic transducer element. When the diaphragm vibrates under the mechanical wave of the acoustic signal, it alters the cavity length of the Fabry–Perot interferometer, leading to a corresponding change in the output optical signal [29]. The basic structure of the EFPI sensor is shown in Figure 1. Typically, to ensure that the reflective surface at the fiber end face remains parallel to the reflective surface of the diaphragm, EFPI sensors employ a multi-sleeve coupling support structure. The effective vibrating radius of the diaphragm is constrained by the supporting sleeve.

Among the various dynamic demodulation techniques for EFPI sensors, quadrature phase demodulation is widely used in high-frequency dynamic signal detection due to its fast response, high sensitivity, and simple system structure [30]. This method selects the static operating point Q within the linear region of the EFPI sensor’s interference spectrum, ensuring the highest detection sensitivity while achieving linear signal output from the EFPI sensor. According to the principle of multiple-beam interference, the output optical signal intensity of the EFPI sensor is given as follows [27]:

(1) $$I\left(\lambda ,l\right)=I_{0}\left(\lambda \right)\cdot \frac{R_{1}+R_{2}-2\sqrt{R_{1}R_{2}}\cos\left(\frac{4\pi nl}{\lambda }\right)}{1+R_{1}R_{2}-2\sqrt{R_{1}R_{2}}\cos\left(\frac{4\pi nl}{\lambda }\right)}$$

In the equation: *I*_0_(*λ*) is the optical intensity incident into the Fabry–Perot cavity of the EFPI sensor; *R*_1_ and *R*_2_ are the reflectivities of the two end faces of the Fabry–Perot cavity; *n* is the refractive index of the medium inside the Fabry–Perot cavity, and when the medium is air, the refractive index is *n* = 1; *l* is the cavity length of the Fabry–Perot interferometer, and *λ* is the wavelength of the optical signal incident into the cavity.

The diaphragm, as the transducing element for both the partial discharge ultrasonic signal and the optical signal, has its vibration sensitivity and natural resonance frequency, which determine the detection sensitivity of the EFPI sensor. Therefore, an analysis of the diaphragm’s dimensions is of particular importance. The structural dimensions of the diaphragm directly influence its static pressure sensitivity and resonance frequency. The first-order natural resonance frequency of a circular quartz diaphragm, fully constrained at its edges, is given as follows [27]:

(2) $$f=\frac{10.21}{2\pi }\sqrt{\frac{Eg}{\left(1-v^{2}\right)\rho }}\cdot \frac{H}{R^{2}}=14.84\sqrt{\frac{E}{\left(1-v^{2}\right)\rho }}\cdot \frac{H}{R^{2}}$$

In the equation, *E* is Young’s modulus of the diaphragm, *ν* is the Poisson’s ratio of the diaphragm, *ρ* is the density of the diaphragm, *H* is the thickness of the diaphragm, and *R* is the effective radius of the circular diaphragm, measured in millimeters (mm); *g* is the acceleration due to gravity.

According to the working principle of the EFPI sensor, changes in the Fabry–Perot cavity length are solely related to the central displacement of the diaphragm. The static pressure sensitivity at the center of the diaphragm is given as follows [27]:

(3) $$S=\frac{3\left(1-v^{2}\right)}{16E}\cdot \frac{R^{4}}{H^{3}}=2.47\times 10^{-6}\frac{R^{4}}{H^{3}}$$

In the equation, the unit of static pressure sensitivity *S* is meters per Pascal (m/Pa).

From Equations (2) and (3), it can be concluded that once the diaphragm material and natural resonance frequency are determined, the larger the *R*/*H* ratio (radius-to-thickness ratio), the greater the diaphragm deformation under the same acoustic pressure. This implies that, for a given diaphragm natural resonance frequency, a thinner diaphragm will have higher static pressure sensitivity. Additionally, the formulas indicate that the gain in static pressure sensitivity from increasing the diaphragm radius is significantly smaller compared to the gain from reducing the diaphragm thickness.

### 2.2. Analysis of Acoustic Coupling Characteristics at the Air-Solid Composite Interface of the Sensor

In the ultrasonic signal detection of partial discharge in large motor stators, EFPI sensors, as acoustic pickup units, can achieve optimal detection performance through efficient acoustic coupling installation methods. Based on the fundamental characteristics of ultrasonic signal propagation in different media, it is known that the damping in gaseous media is high, resulting in rapid sound attenuation, making effective detection difficult. In contrast, solid media can propagate over longer distances compared to gaseous media, primarily because the speed of sound is faster in solid media, wavelengths are longer, and there is no significant viscous damping to cause large-scale attenuation [20]. This favors the diffusion and propagation of ultrasonic signals from partial discharge. Therefore, the EFPI sensor should be coupled to the outer end face of the silicon steel sheet at the stator slot of the large motor to enable effective detection of ultrasonic signals from partial discharge.

**Figure 2:**
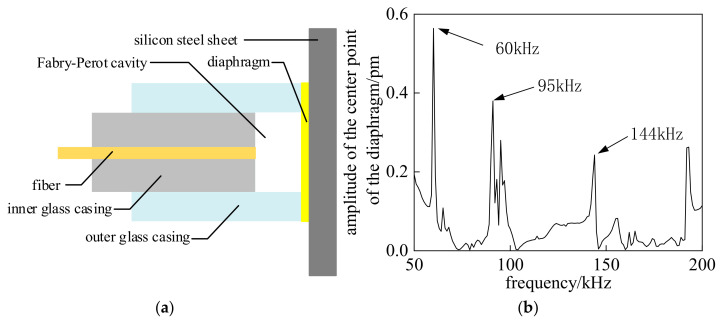
Simulation calculation model and results. (**a**) Simulation calculation model. (**b**) Simulation results.

However, the key issue lies in optimizing the efficient coupling between the dia-phragm-based acoustic-sensitive structure at the front end of the EFPI sensor and the solid silicon steel sheet. Therefore, this study employs the acoustic-solid coupling module in the COMSOL Multiphysics 6.2 simulation software to establish a simulation model and analyze the force coupling mechanism of the EFPI sensor diaphragm at gas-solid composite interfaces. The simulation model is shown in Figure 2a, where the EFPI sensor diaphragm is made of quartz, with a diaphragm thickness of 20 μM, an effective vibrating radius of 0.9 mm, and a natural resonance frequency of 66 kHz. By applying a pressure of 1 Pa on the solid media interface, the amplitude-frequency characteristic curve of the EFPI sensor diaphragm is calculated, with the results shown in Figure 2b.

**Figure 3:**
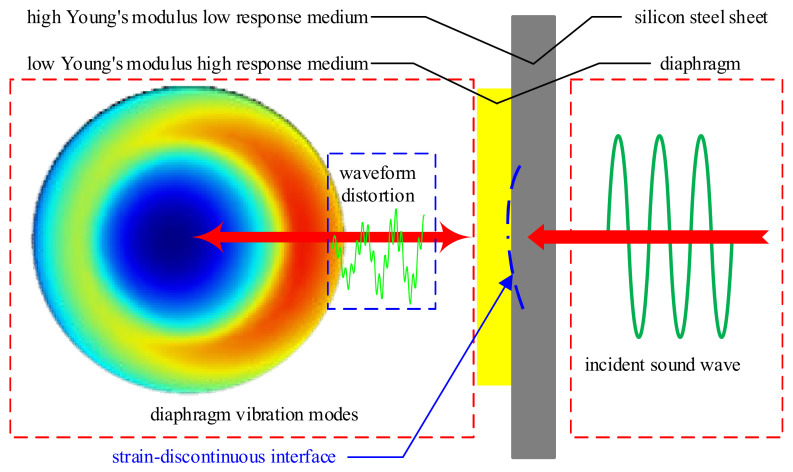
Mode shape distribution and force analysis of the diaphragm.

The calculation results of the amplitude-frequency characteristic curve reveal that the EFPI sensor diaphragm does not exhibit a high-response vibration within its natural resonance frequency range. Instead, it shows vibration responses with small amplitudes across multiple frequency ranges, which is inconsistent with the resonant detection characteristics of the EFPI sensor. The mode shape distribution of the quartz diaphragm at the highest response of 60 kHz, obtained through the simulation model, is shown in Figure 3.

The simulation results reveal that the vibration distribution of the diaphragm does not exhibit a typical regular pattern and does not conform to the forced vibration modes generally observed in circular diaphragms. This is attributed to the principle of mechanical equilibrium, which states that stress is continuous at the interface between two materials. Specifically, at the interface between the quartz diaphragm and the steel plate, the stress is continuous and equal. However, the deformation caused by the same stress applied to two materials with different Young’s moduli differs, as deformation is inversely proportional to Young’s modulus. Consequently, the quartz diaphragm undergoes greater deformation, while the steel plate experiences less.

This disparity results in discontinuity in strain at the interface, creating gaps that lead to stress discontinuity between the quartz diaphragm and the steel plate. As a result, when sinusoidal acoustic signals are transmitted from the steel plate to the quartz diaphragm, a vibration pattern emerges that alternates between continuity and discontinuity. This is particularly evident during the negative half-cycle of the vibration signal, which cannot be fully recovered. The diaphragm loses its sinusoidal vibration characteristics under the applied forces, impairing the EFPI sensor’s ability to leverage its narrowband resonant detection advantage for PD ultrasonic signals. This, in turn, results in reduced detection sensitivity.

**Figure 4:**
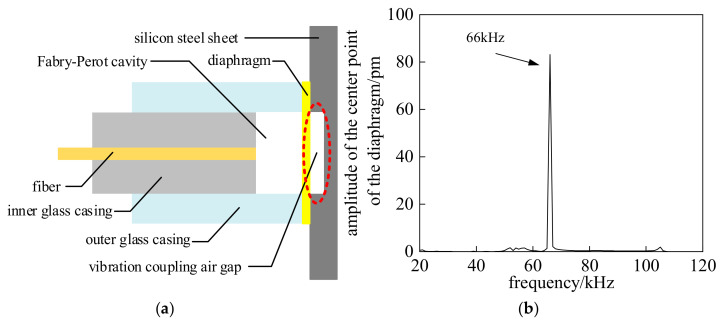
Simulation model and results. (**a**) Simulation model with vibration coupling air gap (**b**) Simulation results.

Considering the above situation, this paper proposes adding a vibration coupling air gap to facilitate sinusoidal vibration of the EFPI sensor diaphragm. This air gap is located at the coupling interface between the diaphragm and the solid medium, and its structure is shown in Figure 4. A simulation model consistent with that in Figure 3 is established to calculate the diaphragm’s amplitude-frequency characteristics, with the results shown in Figure 4b.

A comparison between Figure 2b and Figure 4b reveals that the EFPI sensor diaphragm exhibits a significant high-response vibration at its natural resonance frequency, which is consistent with the analytical solution. In both simulation models, the applied pressure is the same. Under resonant conditions with the vibration coupling air gap, the vibration response amplitude reaches 83.16 pm, which is 148 times the maximum vibration amplitude in the absence of the coupling air gap. Based on the analysis of these results, it can be concluded that the structural design of the vibration coupling air gap enables efficient coupling between the EFPI sensor diaphragm and the solid medium of the silicon steel sheet, allowing for the detection of partial discharge ultrasonic signals via the diaphragm’s narrowband resonant characteristics.

### 2.3. Sensor Fabrication

Since the EFPI sensor utilizes a resonant mode for detecting ultrasonic signals from partial discharge (PD), it exhibits typical narrowband detection characteristics. Its detection bandwidth must align with the primary spectral range of PD ultrasonic signals to achieve optimal detection performance. Therefore, the resonant detection frequency of the EFPI sensor needs to be designed based on the frequency characteristics of PD ultrasonic signals.

The frequency distribution range of ultrasonic signals generated by partial discharge typically falls between 20 kHz and 500 kHz, with different standards and equipment requiring or recommending different detection bandwidths. CIGRÉ (International Council on Large Electric Systems) suggests using a frequency range of 40 kHz to 300 kHz for ultrasonic detection, especially for capturing PD signals in gas-insulated equipment [31,32,33]. Ultrasonic signals within this frequency range effectively reflect the transformation of mechanical energy into sound waves during the partial discharge process. Simultaneously, the IEEE standard recommends a frequency range of 20 kHz to 200 kHz for detecting partial discharge, applicable to other high-voltage electrical equipment, including power transformers [34,35]. Although the frequency range of PD ultrasonic signals is broad and varies to some extent, it is well-established that higher-frequency signals experience greater attenuation. Therefore, for PD ultrasonic signal detection, the resonant detection frequency of the EFPI sensor should be selected in a relatively lower range, ideally within the overlapping low-frequency region recommended by CIGRÉ and IEEE, around 40–50 kHz. However, an important consideration is that the Barkhausen effect in motors and transformers occurs at frequencies close to 50 kHz. This phenomenon is closely related to the characteristics of ferromagnetic materials used in the equipment.

Ferromagnetic materials, such as silicon steel, exhibit complex domain structures, and when subjected to an external magnetic field, domain walls suddenly jump, generating Barkhausen noise. The grain structure, domain size, and defects in silicon steel affect the speed and frequency of domain wall movement. The core materials of motors and transformers are often specially treated and optimized, with rapid domain flipping and high magnetization rates concentrating the typical Barkhausen effect frequency in the tens of kHz range, particularly near 50 kHz. To better avoid the high-frequency uncertainties associated with this effect, the resonant detection frequency of the EFPI sensor should be designed above 60 kHz to minimize potential interference.

**Figure 5:**
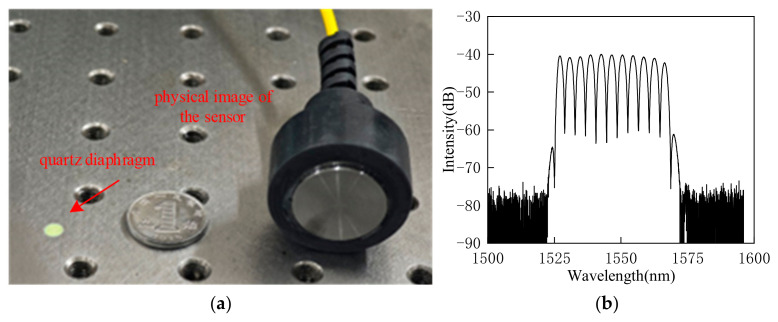
Physical image of the EFPI sensor and its interference spectrum. (**a**) Physical image of the sensor. (**b**) Interference spectrum of the sensor.

According to Equations (2) and (3), the thinner the EFPI sensor diaphragm and the larger its radius, the higher the detection sensitivity and the lower the resonant frequency. Based on the existing fabrication processes for quartz glass diaphragms, a thickness of 20 μM is the standardized production specification for quartz diaphragms. Thus, considering the discussions above, the EFPI sensor diaphragm was designed with a thickness of 20 μM and an effective vibrating radius of 0.9 mm. Under these conditions, the resonant detection frequency of the EFPI sensor is 66 kHz, and the static pressure sensitivity is 0.203 nm/Pa. A schematic and a physical image of the fabricated EFPI sensor are shown in Figure 5a. The quartz capillary glass is supported externally by a 3D-printed sleeve structure, and its interior is filled with epoxy resin. The front-end vibration coupling air gap for acoustic impedance matching with the steel sheet is fabricated using laser etching with a depth of 100 μM and a radius of 0.9 mm. During the fabrication process, the Fabry–Perot cavity of the EFPI sensor is adjusted using a three-axis micro-positioning platform. The interference spectrum of the fabricated EFPI sensor is shown in Figure 5b.

**Figure 6:**
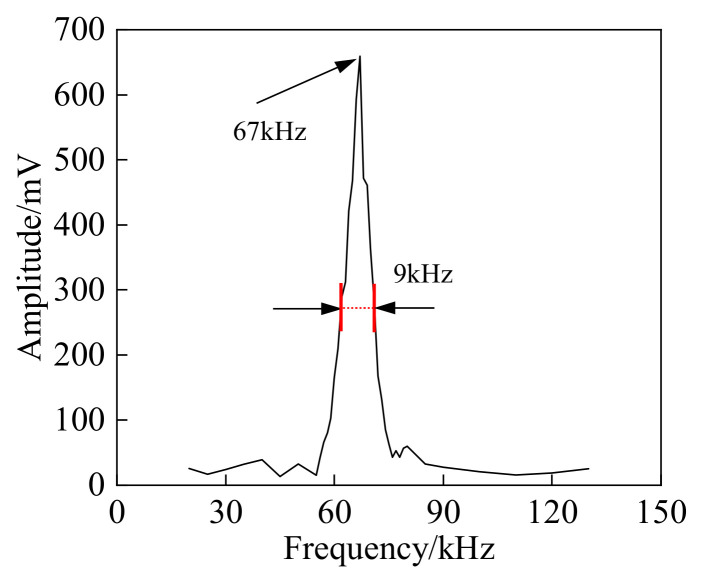
Frequency response characteristic curve of the EFPI sensor.

The amplitude-frequency characteristics of the fabricated EFPI sensor were tested using a calibrated acoustic emission PZT sensor. The test results are shown in Figure 6. The resonant detection frequency of the EFPI sensor is 67 kHz, with a deviation of 1 kHz from the designed frequency of 66 kHz, which falls within the acceptable range. Additionally, the amplitude-frequency characteristics of the EFPI sensor indicate that the detection bandwidth is approximately 9 kHz, which is characteristic of a typical narrowband resonant detection mode.

## 3. Distribution Characteristics of Partial Discharge Ultrasonic Signals in the Stator and Location of Discharge Points

### 3.1. Analysis of Ultrasonic Signal Distribution Characteristics in Stator Slots

Within large electric motor stators, particularly inside the stator slots, there are numerous composite interfaces and a narrow, elongated structure. The stator slots contain interfaces of different materials (such as copper wire and insulation materials), which significantly affect the reflection, refraction, and attenuation of sound waves. Additionally, the narrow structure of the stator slots leads to multiple reflections and diffractions of sound waves during propagation, complicating the sound wave paths. Because sound waves travel through different paths at varying times, waves from multiple paths can overlap or interfere at certain points, creating a complex sound field distribution. This sound distribution cannot be directly quantified through theoretical analysis due to its intricate nature; however, understanding the sound field distribution characteristics of ultrasonic signals from partial discharge (PD) at the end of the stator slots can directly inform the selection of installation positions for the EFPI sensor.

**Figure 7:**
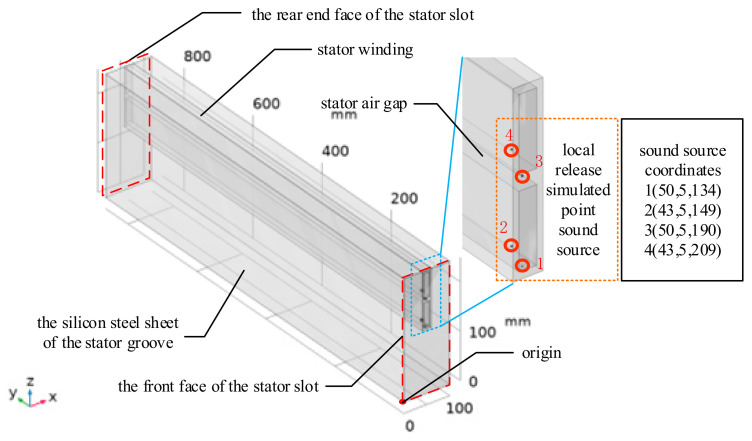
Simulation model for ultrasonic signals from partial discharge in the stator.

In this study, COMSOL Multiphysics 6.2 simulation software is utilized to investigate the ultrasonic signals from PD in the stator slots. Given that the structure of large electric motor stators is periodically arranged, a simplified analysis can be performed. The acoustic propagation characteristics within a single stator slot provide insights into the sound distribution and propagation characteristics of PD ultrasonic signals in the stator structure. The simulation model is shown in Figure 7.

Using COMSOL Multiphysics 6.2 simulation software, a simulation model was established, employing the Acoustic-Solid Interaction (Frequency Domain) calculation module. The static distribution characteristics of partial discharge ultrasonic signals were simulated by applying the corresponding physical fields and boundary conditions. The ultrasonic waves generated by partial discharge (PD) within the motor stator windings influence the surrounding medium in the form of sound pressure. This process can be expressed as follows [36]:

(4) $$\frac{1}{\rho c^{2}}\frac{\partial^{2}p_{t}}{\partial t^{2}}+\nabla \cdot (-\frac{1}{\rho }(\nabla p_{t}-q_{d}))=Q_{m}$$

Here, *ρ* represents the air density, *c* denotes the speed of sound, *p_t_* indicates the sound pressure magnitude, ∇*p_t_* represents the sound pressure gradient, and *Q_m_* accounts for the attenuation of sound waves in the environment. *q_d_* can represent the impact of the sound source on the external environment, that is, the effect produced when ultrasonic loading is transmitted to different media.

The boundary conditions for the fluid-solid coupling interface are expressed as follows:

(5) $$-n\cdot (-\frac{1}{\rho }(\nabla p_{t}-q_{d}))=0$$

Here, *n* represents the unit normal vector of the boundary, oriented from the fluid to the solid. According to solid mechanics, the effect of the acoustic signal on the solid medium can be expressed as follows:

(6) $$\rho \frac{\partial^{2}u}{\partial t^{2}}=\nabla \cdot S+F_{v}$$

where *u* is the displacement vector of the solid structure, *S* is the stress tensor, and *F_v_* is the body force vector. Once the displacement vector *u* is obtained, further information, such as stress and strain in the solid medium, can be derived.

In the model, the silicon steel sheets and the stator windings are treated as a unified, simplified structure. The dimensions of the stator silicon steel sheet model are as follows: width of 100 mm, height of 250 mm, and length of 865 mm. The stator slot has a width of 20 mm and a height of 120 mm, while the length of the stator winding is consistent with that of the silicon steel sheet model, with a width of 12 mm and a height of 50 mm. The locations of the partial discharge sound source points set in the model are indicated in the figure above, all positioned near the ends of the stator windings, which are common locations for PD occurrences in generator stator windings.

**Figure 8:**
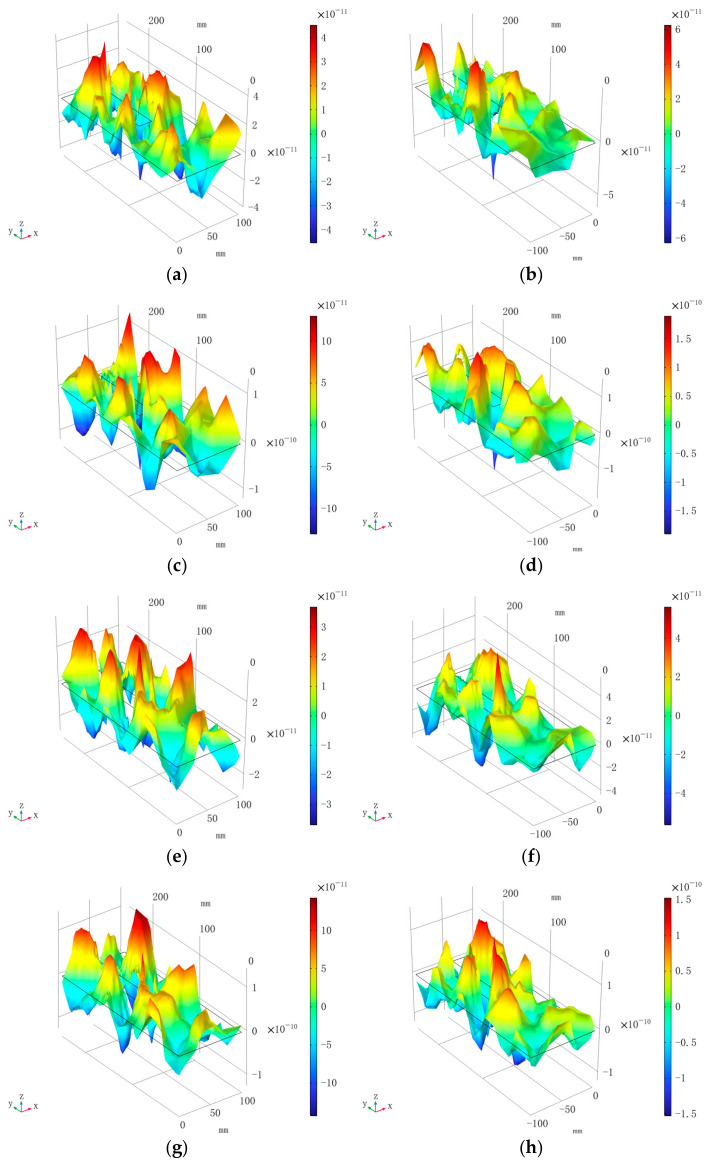
Deformation of the end faces of the stator slot caused by different partial discharge points. (**a**) Deformation of the front end face of the stator slot caused by discharge point 1. (**b**) Deformation of the rear end face of the stator slot caused by discharge point 1. (**c**) Deformation of the front end face of the stator slot caused by discharge point 2. (**d**) Deformation of the rear end face of the stator slot caused by discharge point 2. (**e**) Deformation of the front end face of the stator slot caused by discharge point 3. (**f**) Deformation of the rear end face of the stator slot caused by discharge point 3. (**g**) Deformation of the front end face of the stator slot caused by discharge point 4. (**h**) Deformation of the rear end face of the stator slot caused by discharge point 4.

Since the resonant detection frequency of the EFPI sensor designed in this study is 67 kHz, this frequency is used as the simulated sound source frequency for the simulation model, with a sound pressure amplitude of 1 Pa. Figure 8 illustrates the sound field distribution at the ends of the stator slots for various partial discharge points. The unit for the deformation in the color bar is millimeters (mm).

Comparative analysis reveals that the regions of strain concentration due to ultrasonic signals from partial discharge are located at the edges of the stator slots, particularly near the discharge points. This is attributed to the formation of two acoustic reflection boundaries at the edges of the end faces of the stator windings and stator slots, which create physical conditions conducive to wave superposition and enhancement, leading to strain concentration. Therefore, in practical detection, it is advisable to install the EFPI sensor at the edge of both end faces of the stator slots to achieve optimal detection performance.

### 3.2. Detection and Localization Analysis of Ultrasonic Signals from Partial Discharge in the Stator

**Figure 9:**
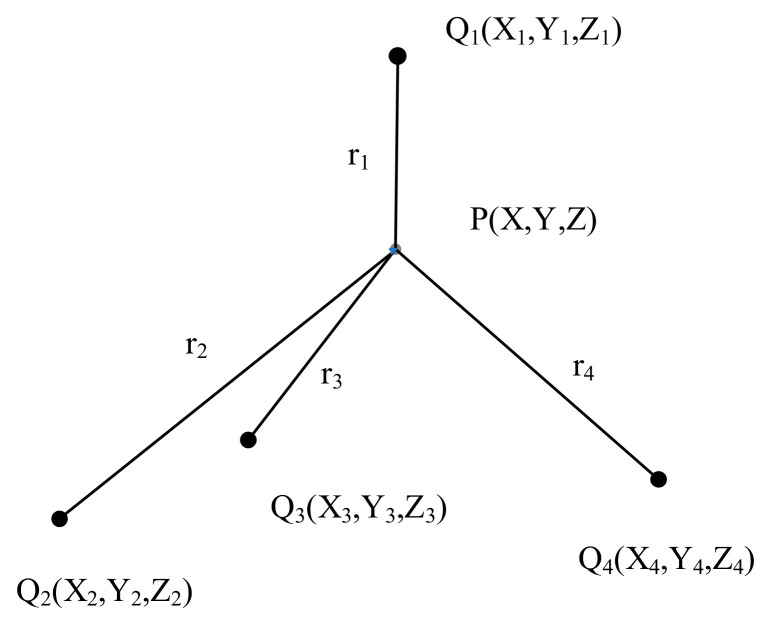
Geometric principle of the Time Difference of Arrival method.

The Time Difference of Arrival (TDOA) method, also known as hyperbolic localization, utilizes the time differences of the signal’s arrival at multiple observation points to determine its location. By employing data from at least four observation points, the position of the partial discharge source in three-dimensional space can be accurately localized. This is achieved by establishing a system of three equations to obtain specific data. The geometric principle underlying this method is illustrated in Figure 9, where P represents the position of the sound source and Qi(xi, yi, zi) denotes the positions of the observation points.

Let the time taken for the ultrasonic signal to travel from the sound source P to the observation point Q_i_ be denoted as *t_i_*. The distance *r*_*i*_ from point P to Q_i_ can be expressed as follows:

(7) $$r_{i}=\sqrt{{\left(x-x_{i}\right)}^{2}+{\left(y-y_{i}\right)}^{2}+{\left(z-z_{i}\right)}^{2}}=v\cdot t_{i}$$

where *v* represents the propagation speed of the signal in the medium.

**Figure 10:**
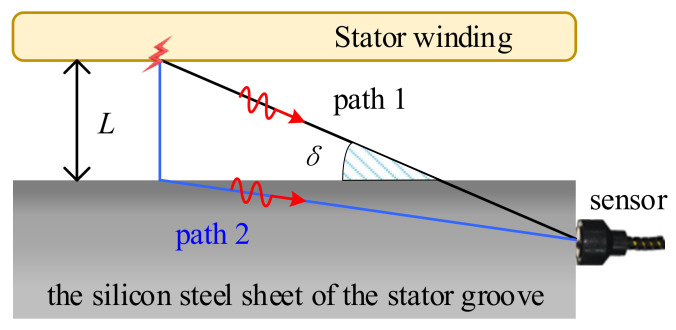
Acoustic path diagram of typical ultrasonic signals from partial discharge reaching the sensor.

When partial discharge occurs in the stator winding, the ultrasonic signal first travels through the air gap before entering the silicon steel sheet and finally reaching the sensor collection point. At this point, there are two possible paths for the ultrasonic signal emitted from the discharge source to reach the EFPI sensor. The first path involves the sound signal propagating directly through the air to the EFPI sensor. The second path involves the signal traveling through the air, impacting the stator slot, and then propagating to the EFPI sensor, as illustrated in Figure 10.

In the localization calculation for the partial discharge source, the first peak is considered the time reference for the sensor’s reception. Although path 1 has the shortest distance, the speed of sound in the medium for path 1 is slower than that in the second segment of path 2, which is in a medium with a higher sound speed. Consequently, it is possible for the ultrasonic signal traveling along the longer path 2 to arrive at the EFPI sensor first. Whether this phenomenon occurs is evidently determined by the distance *L* from the sound source to the stator slot wall and the angle *δ* as depicted in Figure 10. For instance, using the maximum stator slot size of 20 mm in the experimental model adopted in this study, when the angle *δ* is not less than 3.7°, path 2 is the first route for the partial discharge ultrasonic signal to reach the EFPI sensor. It is clear that, in most cases, the partial discharge ultrasonic signal propagates to the EFPI sensor via path 2. Consequently, when employing the time difference of arrival (TDOA) method for localization, it is necessary to consider the distance the discharge source travels to the stator slot wall, thus adding an unknown variable to the TDOA calculation equations, significantly increasing the complexity of solving the equations. However, since the thickness of the air gap in the stator slot is relatively thin, generally only a few millimeters, this distance is considerably smaller compared to the propagation length of sound through the silicon steel sheet and its effect on the arrival time of the sound signal at the sensor can be neglected. Therefore, in this study, the sound speed is uniformly selected as that of the silicon steel sheet, which is 4503.2 m/s.

**Figure 11:**
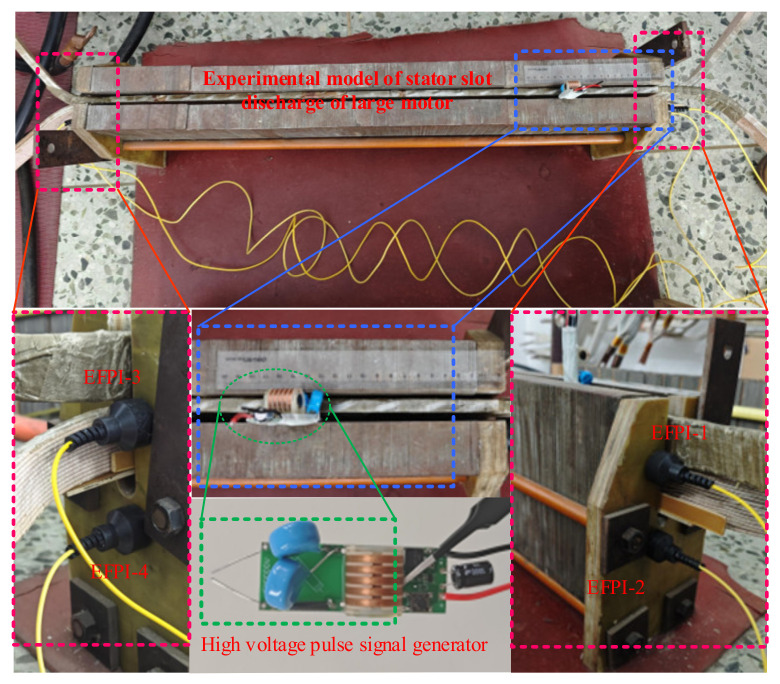
Experimental system diagram.

**Figure 12:**
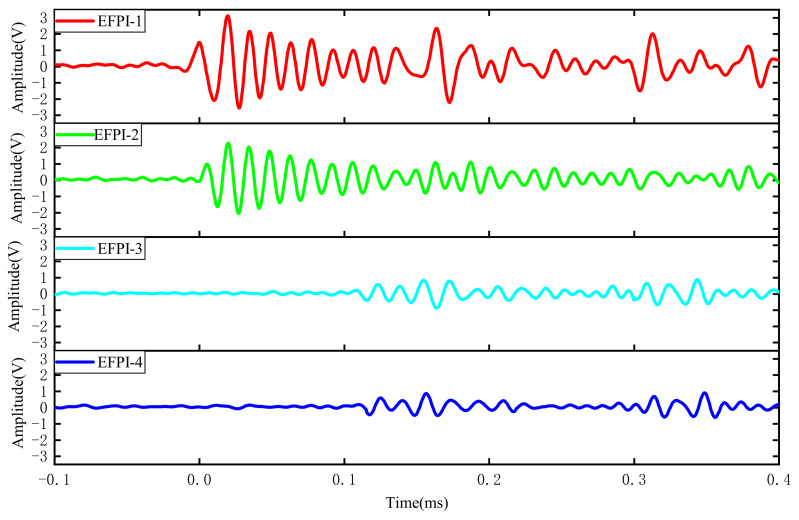
Time domain diagram of stator slot local discharge ultrasonic signal.

This study establishes a physical experimental model for investigating partial discharge in the stator slot. Based on the concentrated strain distribution area of the ultrasonic signals at the stator slot end face, the arrangement of the EFPI sensors is determined. A high-voltage pulse signal generator simulates the actual ultrasonic signals from partial discharge during the experiments. The experimental setup for the partial discharge system is illustrated in Figure 11, with the sound source of the partial discharge located near the end of the stator winding. The experimental results obtained from the four EFPI sensors are shown in Figure 12.

The experimental results indicate that the output signal amplitude of the EFPI-1 and EFPI-2 sensors, which are located closer to the partial discharge source, is significantly higher than that of the EFPI-3 and EFPI-4 sensors, consistent with the actual situation. Moreover, the EFPI-1 sensor is the first to receive the ultrasonic signal from the partial discharge, marking the time of signal activation as zero. The time differences between the other sensors and the EFPI-1 sensor, along with the coordinates of the four sensors in the experimental model, are presented in Table 1.

In the experimental study, the coordinates of the partial discharge simulation point were (45, 150, 180). Based on the results obtained, the calculated position of the partial discharge point is (40.5, 149.7, 181.4). Ignoring the influence of non-uniform medium sound velocity, the TDOA method was used for calculation, and the linear deviation between the partial discharge point and the preset position of the model was 4.73 mm. In order to verify that the influence of non-uniform medium sound velocity was ignored, the local discharge source was shifted along the z-axis, and four groups of partial discharge experiments were carried out. The experimental results are shown in Table 2.

According to the experimental results can be found, with the partial discharge point moving along the z-axis, δ gradually becomes smaller. When the influence of the sound velocity of the inhomogeneous medium is ignored, the accuracy of the location calculation directly using the TDOA method is higher. When the partial discharge simulation point is located in the middle of the model, the positioning error is the smallest, and the error value is 2.78 mm. The results of the positioning calculation meet the requirements of engineering inspection.

## 4. Conclusions

This study designed an EFPI sensor front-end membrane packaging structure with a vibration-coupling air gap based on multi-beam interference theory and the characteristics of fluid–solid coupling mechanical systems. This design facilitates the direct adhesive installation of the EFPI sensor on the end face of large motor stator slots. Furthermore, COMSOL Multiphysics 6.2 simulation software was utilized to analyze the distribution characteristics of ultrasonic signals from partial discharges on the end face of the stator slots, identifying an optimal installation location for the EFPI sensor to efficiently detect partial discharge ultrasonic signals. Subsequently, a physical experimental model was established to verify the feasibility of wall-adhered EFPI sensors in detecting partial discharge ultrasonic signals and the accuracy of locating the discharge points. The conclusions are as follows:

1. To leverage the narrow-band resonant detection sensitivity of the EFPI sensor, the front-end packaging structure was designed with a vibration-coupling air gap, enabling full-wave periodic vibrations of the sound-sensitive membrane. This design ensures effective vibrations in the resonant state and high sensitivity in detection, with a resonant frequency of 66 kHz, consistent with theoretical calculations;

2. Following partial discharge in the stator windings, the strain distribution on both end faces of the stator slots caused by the partial discharge ultrasonic signals is primarily concentrated at the edges of the stator slots when the EFPI sensor operates at the 66 kHz resonant detection frequency;

3. In the detection of partial discharge ultrasonic signals, all four EFPI sensors effectively detected the signals. By neglecting the influence of the air gap in the stator slot on the propagation path of the partial discharge ultrasonic signals, the TDOA method was employed to locate the discharge point. The maximum positioning error is 4.73 mm, and the minimum positioning error is 2.78 mm, which meets practical engineering requirements.

## Figures and Tables

**Table 1:** Sensor coordinate positions in the experimental model.

Sensor Number	Sensor Coordinate (mm)	Time Difference Between Sensor and EFPI-1 Sensor (ms)
1	(30, 0, 150)	0
2	(30, 0, 225)	0.00804
3	(30, 865, 150)	0.12484
4	(30, 865, 225)	0.12450

**Table 2:** Experimental results of partial discharge location.

Experimental Group	Actual Location of the Partial Discharge Point (mm)	Calculated Position of Partial Discharge Point (mm)	Positioning Error Value (mm)
1	(45, 150, 180)	(40.5, 149.7, 181.4)	4.73
2	(45, 150, 240)	(41.4, 151.5, 239.6)	3.92
3	(45, 150, 300)	(43.2, 148.2, 301.8)	3.12
4	(45, 150, 360)	(43.3, 147.8, 359.2)	2.89
5	(45, 150, 400)	(43.9, 148.9, 402.3)	2.78

## Data Availability

The original contributions presented in the study are included in the article; further inquiries can be directed to the corresponding author.
